# The Accelerated Onset of Calciphylaxis in a 72-Year-Old Female Hemodialysis Patient

**DOI:** 10.7759/cureus.58492

**Published:** 2024-04-17

**Authors:** Agostino Grittani, Konstantinos Kouzounis, Samantha Zarry, Jose H Suarez

**Affiliations:** 1 Internal Medicine, St. George's University, St. George's, GRD; 2 Plastic Surgery, St. George's University, St. George's, GRD; 3 Internal Medicine, The University of Western Ontario, Toronto, CAN; 4 Family Medicine, Keralty Hospital, Miami, USA

**Keywords:** calcific uremic arteriopathy, anemia in ckd, hemodialysis complication, end stage renal disease (esrd), calciphylaxis

## Abstract

Calciphylaxis is a unique medical condition characterized by calcification of the medial layer of arterioles and soft tissues in a patient’s skin at the level of the dermis and subcutaneous adipose tissue. The rate of progression of calciphylaxis is rapid, starting with a reduction of blood flow that leads to ischemic changes in the skin that can manifest as painful cutaneous erythematous nodules or plaques and later as skin ulceration. The majority of patients affected by calciphylaxis have predisposing comorbidities such as end-stage renal disease with a long history of hemodialysis and electrolyte abnormalities in calcium, phosphate, and parathyroid hormone levels. This report presents the case of a 72-year-old female patient on hemodialysis who developed calciphylaxis. The methods for early prognosis (the methods of early diagnosis), including clinical presentation, risk factors, imaging techniques, and laboratory investigations, are discussed. The presented case is particularly noteworthy given the onset of calciphylaxis within a mere three months of initiating hemodialysis, a timeline significantly shorter than the typically observed period in most patients. (The case detailed in this report outlines the rapid onset of calciphylaxis in a patient who was receiving hemodialysis for only three months.) This patient with early-onset calciphylaxis highlights the unpredictable nature of calciphylaxis and the need for increased clinical vigilance even in the initial stages of hemodialysis.

## Introduction

Calciphylaxis, or in this case, calcific “uremic” arteriolopathy, is a rare and life-threatening condition, primarily manifesting in patients with end-stage renal disease (ESRD). Its pathogenesis is marked by medial calcification of arterioles, leading to ischemia, necrosis, and the development of nonhealing ulcers. Understanding the risk factors that predispose patients to this condition and monitoring the clinical features of calciphylaxis as it progresses is crucial, despite its rarity. Patients with calciphylaxis have a 2.5-3 times higher mortality rate than patients without this condition on hemodialysis, while the first-year mortality rate for calciphylaxis is estimated to be as high as 45-80% of patients [1-4].

The report further delves into methods for early prognosis, a crucial aspect of patient care that can positively impact patient outcomes. It reviews the role of clinical presentation, imaging techniques, and laboratory investigations such as electrolyte or hormonal abnormalities in diagnosing the condition, as early detection is pivotal in managing disease progression and improving patient survival. Special attention is paid to the interplay between secondary hyperparathyroidism, abnormalities in calcium, phosphate, and vitamin D metabolism, and their contribution to the pathogenesis of calciphylaxis in hemodialysis patients, emphasizing the need for comprehensive multidimensional patient care and monitoring [1-6].

## Case presentation

A 72-year-old female patient with a history of ESRD secondary to type II diabetes mellitus undergoing hemodialysis for the past three months presented with painful necrotic ulcers in the lower extremities. She reported a three-week history of progressive pain, pruritus, and erythema, which progressed mainly to necrotic ulcers on the medial aspect of her right leg.

The patient had a past medical history of ESRD on hemodialysis three times a week, paranoid schizophrenia, diabetes mellitus type 2, hypertension, and anemia. She was alert, awake, and oriented x3 but confused and a poor historian. Her medical history was corroborated via a phone call with her husband. Per the patient’s husband, the lesion on her lower extremities was provoked due to skin picking associated with schizophrenia and “fluid retention,” making her skin open and infected. The patient had no prior history of trauma, such as burns or fractures in the area of skin ulceration.

Lower extremity radiographs were obtained and demonstrated extensive unilateral calcifications in the right lower limb. Computed tomography angiography (CTA) of the abdominal aorta and lower extremities found middle and distal right anterior tibialis and posterior tibialis artery occlusion, with atherosclerotic changes and soft tissue ulceration and edema. A punch biopsy was completed after CTA and confirmed the calcified areas in the right medial leg.

On admission to the emergency room, the vital signs recorded are seen in Table 1, and the concurrent laboratory investigations taken on admission are seen in Table 2.

**Table 1:** Patient vitals upon admission to the emergency room BPM, beats per minute

Vital signs recorded	Recorded values on admission
Blood pressure	171/71 mmHg
Pulse	79 BPM
Respirations	20/min
Temperature	98.6 °F
SpO2	99% on room air

**Table 2:** Initial laboratory investigation values on CBC and CMP BUN, blood urea nitrogen; CBC, complete blood count; CMP, complete metabolic panel; GFR, glomerular filtration rate

CBC and CMP findings	Recorded values	Reference range
White blood cell count	12,600 cells/µL	4,500-11,000/mm
Hemoglobin	8.8 g/dL	Female: 12.0-16.0 g/dL
Hematocrit	27.90%	Female: 36-46%
Platelets	449,000 cells/µL	150,000-400,000/mm^3^
Glucose	166 mg/dL	64-100 mg/dL (3.55-5.55 mmol/L)
BUN	23 mg/dL	6-20 mg/dL (2.14-7.14 mmol/L)
GFR	8 mL/min/1.73m^2^	90-120 mL/min/1.73 m^2^
Creatinine	5.4 mg/dL	0.8-1.2 mg/dL
Ca2+ (serum)	8.7 mg/dL	8.5-10.2 mg/dL

The urinary analysis obtained was unremarkable, and her lactic acid was within normal limits.

**Figure 1:**
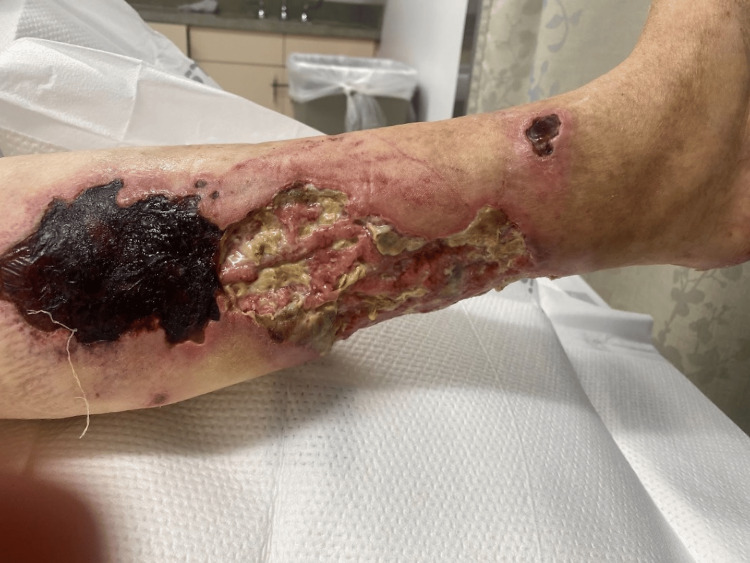
Right leg ulcer

Physical examination revealed multiple painful, nonhealing, necrotic ulcers on the lower extremities, predominantly in the medial aspect of her right leg and partially in the thighs (Figure 1).

## Discussion

Early physical exam signs and risk factors

Patients on long-term hemodialysis, like the present case, were found to have a higher risk of developing calciphylaxis due to the interplay between ESRD, secondary hyperparathyroidism, and abnormalities in calcium, phosphate, and vitamin D metabolism [7,8]. The likely association between the abnormalities in these given hormones and electrolytes suggests that as the materials needed for vascular calcification increase, more calcifications happen in the body [7,8]. Evidence suggests that patients receiving hemodialysis who have chronic hypertension, diabetes, and phosphate or calcium electrolyte derangement will have vascular calcification 83% of the time visible on plain radiographs [7].

Early diagnosis of calciphylaxis is challenging due to its nonspecific clinical presentation [9]. However, specific risk factors such as female sex, obesity, diabetes, and warfarin use have been identified [3,10]. The mechanism of action for why these risk factors contribute to calciphylaxis is poorly understood, but it is currently thought that these risk factors increase the likelihood of endothelial injury and reduced blood flow. It is suggested that these vascular events are triggers that contribute to blood stasis, the formation of microthrombi, and luminal narrowing leading to tissue ischemia, necrosis, and ulceration [4,10].

A physical examination showing painful skin lesions, livedo reticularis, and subcutaneous nodules, as in our patient, may suggest calciphylaxis [11]. Serum calcium and phosphate levels, parathyroid hormone levels, and alkaline phosphatase are commonly seen as abnormal in patients with calciphylaxis [6]. The monitoring of these metabolic markers can assist in the early diagnosis of uremic calciphylaxis, but it is important to note that similar abnormal electrolyte levels are also commonly seen in patients with chronic kidney disease who do not develop calciphylaxis. Additionally, clinical data has shown that calciphylaxis, a nonuremic subtype, occurs in patients with electrolyte levels within normal limits, adding to the difficulty of calciphylaxis diagnosis and monitoring [11].

Early establishment of a baseline for the risk factors associated with calciphylaxis can help establish a starting point for preventative treatments, if indicated. Patients who have a history of progressive ESRD with electrolyte abnormalities and obesity should be counseled on the potential lifestyle changes and medical reconciliations that can be done to prevent potential calciphylaxis onset.

Diagnostic modalities

Despite being invasive, a skin biopsy provides the most definitive diagnosis [12]. Nevertheless, given the risk of nonhealing biopsy sites in calciphylaxis, noninvasive imaging techniques like plain radiographs, computed tomography, and magnetic resonance imaging have been proposed for early diagnosis [13-16].

Three-phase bone scintigraphy showing increased uptake in soft tissues is a relatively new imaging modality that can suggest calciphylaxis in the appropriate clinical context [17].

Multidimensional treatment plans

The management of calciphylaxis includes wound care, pain management, controlling the calcium-phosphate product, and discontinuing any vitamin K antagonist [9]. Although off-label, sodium thiosulfate (STS) has shown promise in treating calciphylaxis due to its potential calcium-chelating and antioxidant properties [18].

Patients on STS showed initial improvement with decreased pain and a halting of the progression of skin lesions. Yet, due to the critical nature of calciphylaxis, close follow-up is warranted.

The therapeutic strategy for calciphylaxis aims to control its risk factors and manage its severe clinical manifestations. The initial focus is on addressing the elevated calcium-phosphorus product and discontinuing any vitamin K antagonists that could potentially exacerbate the disease [9]. Pain management and wound care also form an integral part of treatment to improve the patient’s quality of life.

The prognosis, however, remains guarded. The mortality rate associated with calciphylaxis is exceedingly high due to complications like sepsis and organ failure. Despite initial improvement, our patient’s condition warrants rigorous monitoring and follow-up for a comprehensive assessment of effective treatment and potential complications.

## Conclusions

Calciphylaxis remains a diagnostic challenge with a high mortality rate. Early recognition and management are critical. This case highlights the importance of heightened clinical suspicion and the utilization of appropriate diagnostic modalities to ensure timely diagnosis and management.

Indeed, the case presented here is unusual and of great interest due to the relatively short duration of hemodialysis before the onset of calciphylaxis. While the disease typically develops in patients with prolonged periods of ESRD and extended durations on hemodialysis, our patient developed calciphylaxis after three months of treatment. This suggests that other contributing factors, such as the patient’s age, gender, and underlying diabetes mellitus, may have accelerated the onset of this condition. It highlights the unpredictable nature of calciphylaxis and the necessity for a high degree of clinical suspicion even in the relatively early stages of hemodialysis. This unusual timing further underlines the need for ongoing research into calciphylaxis’s pathogenesis and risk factors. A deeper understanding of why some patients develop the condition earlier than others could improve prevention strategies, early diagnosis, and treatment, ultimately enhancing the prognosis for patients at risk of this devastating disease.
